# ^226^Ra, ^232^Th and ^40^K contents in water samples in part of central deserts in Iran and their potential radiological risk to human population

**DOI:** 10.1186/2052-336X-12-80

**Published:** 2014-05-01

**Authors:** Elham Ehsanpour, Mohammad Reza Abdi, Mojtaba Mostajaboddavati, Hashem Bagheri

**Affiliations:** 1 Department of Physics, Faculty of Science, University of Isfahan, Isfahan, Iran; 2 Department of Nuclear Engineering, Faculty of Advanced Sciences & Technologies, University of Isfahan, Isfahan, Iran; 3 Department of Geological Science, Faculty of science, University of Isfahan, Isfahan, Iran

**Keywords:** Activity concentration, Gamma spectrometry, Water, Anarak-khour, Iran

## Abstract

**Background:**

The radiological quality of ^226^Ra, ^232^Th and ^40^K in some samples of water resources collected in Anarak-Khour a desertic area, Iran has been measured by direct gamma ray spectroscopy using high purity germanium detector in this paper.

**Result:**

The concentration ranged from ≤0.5 to 9701 mBq/L for ^226^Ra; ≤0.2 to 28215 mBq/L for ^232^Th and < MDA to 10332 mBq/L for ^40^K. The radium equivalent activity was well below the defined limit of 370Bq/L. The calculated external hazard indices were found to be less than 1 which shows a low dose.

**Conclusion:**

These results can be contributed to the database of this area because it may be used as disposal sites of nuclear waste in future.

## Background

The presence of naturally occurring radionuclides as well as some elements provides important information about the quality of water resources especially drinking water [1].

Naturally occurring radioactive materials (NORM) consist of uranium, thorium, potassium and any of their decay products such as radium and radon. Concentrations of these natural radioactive elements are very low in the earth’s crust and atmosphere. These elements can be brought to the surface by human activities. Although the radioactive elements in the earth’s crust are the reasons of presence of radioactivity in water resources, high concentration of radioactive materials in water resources might be accidentally or intentionally [2,3]. The public can be affected by the environment where is adjacent to the released point of the radioactive materials [4]. If radioactive materials are released into the environment, radionuclides may be moved into the body by inhalation and ingestion, which causes internal exposure. Fakeha et al. analyzed samples from well water and bottled drinking water from the Western Province of Arabia for concentrations of natural radioactivity and their contribution to the absorbed dose from water samples using gamma spectroscopy method [1]. Fasunwon et al. studied the activity concentrations of natural radionuclide levels in well waters of Ago Iwoye, Nigeria by HPGe (high purity germanium) spectrometer [5]. They estimated that radiological health burden on the human populace is very minimal and has neither health implications nor affect the background ionization radiation. In a research article studied that natural radioactivity of different brands of commonly sold bottled drinking water in the federal capital Islamabad and Rawalpindi city of Pakistan and found mean concentrations of ^226^Ra, ^232^Th and ^40^K were 11.3 ± 2.3, 5.2 ± 0.4 and 140.9 ± 30.6 mBq/L, respectively using gamma spectroscopy technique [6].

The United Nations Scientific Committee on the Effects of Atomic Radiation (UNSCEAR) estimated that exposure to natural radionuclides contributes around 70% of the population radiation dose. The global average human exposure to natural sources is 2.4mSv/y and the weight of water and food is about 0.3mSv/y.

The objective of this study is to obtain a representative estimate of the concentration levels of natural radionuclides in water resources which might be used as drinking water in some our studied sites from the central deserts of Iran, also estimate the corresponding radiation doses for people consuming this water. With water analysis, soil analyses have been performed in this area. The obtained results can be contributed to the baseline data of radionuclide concentrations in this area.

### Description of the study area

**Figure 1:**
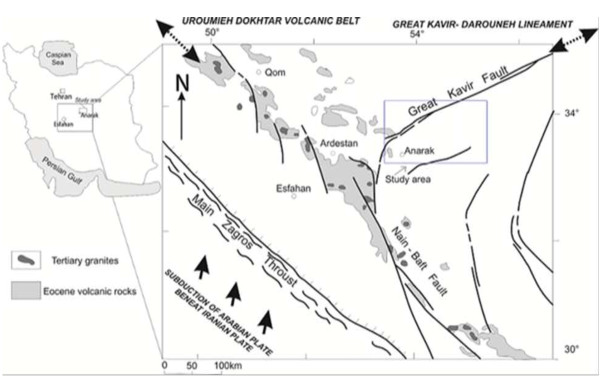
**Main structural lineaments in Central Iran and location of the study area (modified from **[8]**).**

The intersection of Uroomieh-Dokhtar Magmatic Belt (UDMB) and the major Great Kavir- Doruneh fault (GKDF) are two dominant structural features in the Anarak-Khour area and the change of direction of Great Kavir-Doruneh fault towards the Nain-Baft fault (Figure 1). This region occupies the north-western corner of the Central-Eastern Iranian microplate. This terrane is an approximately 2300km^2^ region of moderate relief surrounded by folding and thrust belts within the Alpine-Himalayan orogenic system of western Asia. This terrene is an area of continuous continental deformation in response to ongoing convergence between the Arabian (Gondwana) and Turan (Eurasian) plates [7]. Continental volcanism along the UDMB and in Central Iran, which also comprises the volcanic rocks of the Anarak-Khour area, is attributed to that subduction.

In the Anarak-Khour area there are a few compositionally complex hydrothermal Cu-Ni-Co deposits which always interested for researchers. Apart from Cu, Ni and Co the ores contain As and U and occasionally Pb, Zn, Au and Ag. All these deposits are localized in the same area under similar geological environment along the north-western and western surroundings of Anarak-Khour massif (Figure 1). These deposits contain a distinctive set of elements and minerals. In the Anarak area, Co, Ni and As are abundant but there is little Ag or Bi [8]. The deposits also show some U. Cu is different because its concentration is high and in particular the abundance of copper arsenides. As with other deposits, Fe is present in only small amounts and S is rare in the arsenide stage of mineralization. Talmessi and Meskani mines are ancient mines for Cu, Ni and Co products that mining activities have ceased since 1960. Recently, exploration activities were conducted by the atomic energy organization of Iran in the course of uranium exploration but there is not any U mine in this area until now. The most important active mine in the area is Nakhlak lead deposit, 40 Km east of Anarak. In geology and geochemistry, the radioactive deposits are associated with high concentrations of heavy metals such as As, Cd, Co, Cr, Cu, Fe, Hg, Ni, Pb, S, Sb and Zn. It seems the presence of deposits and location of this area along the fault is caused these materials coming from the deeper layers to the surface layers and this can be a reason for founding the radionuclides and assessing the radiological risk in this area.

### Experimental

#### ***Sampling***

**Figure 2:**
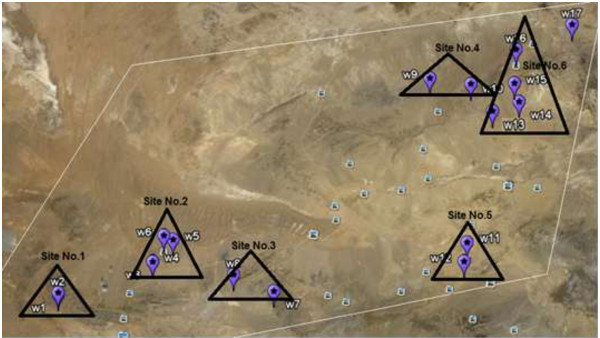
Sampling sites.

Anarak-khour is a landscape that is very dry because of low rainfall amounts (precipitation) then finding water in this area is difficult. Water sampling was taken from well water, reservoir and ground water. The sample locations were recorded in terms of degree-minute-second latitudinal and longitudinal position using a hand-held Global Positioning System (GPS) unit in Table 1. Places that would likely have a greater radioactivity concentration were selected as sample sites. Then the water samples of this area are collected from 33°22’46.52” N (with 53°27’47.90” E) to 34°12’25.12” N (with 55°18’55.40” E). The studied area is located within the rectangular area and is divided into six sites as shown in Figure 2. Reference methods for collecting procedure and handling of the water samples were taken from the International Atomic Energy Agency (IAEA, water sampling and laboratory treatment). Some these samples came from wells with submerged pumps. The water samples were gathered in two liter plastic bottles and were acidified with nitric acid to pH < 2. Physicochemical parameters of water such as temperature and pH were also measured in order to find the impact of these parameters on the concentration of the radionuclides in water. For water samples 800 mL of each sample was transferred to a Marinelli beaker. Then, the Marinelli-beaker was sealed and kept for at least 5 weeks. During this time, the daughter of radon achieves to equilibrium with ^226^Ra. Then the samples were ready to analysis by gamma spectroscopy [9].

**Table 1:** Sampling spots information

**Longitude**		**Latitude**	**Altitude**	**Temperature**	**pH**	**Characteristic**
**Site no.1 (Talmessi mine)**						
1	33°22’46.52” N	53°27’47.90” E	1465	13	8	Ground water
2	33°23’9.02” N	53°27’47.16” E	1476	13	8.1	Ground water
**Site no.2 (Nakhlak mine)**						
3	33°29’0.12” N	53°48’6.19” E	1134	21	8.39	Well water
4	33°29’5.23” N	53°48’2.69” E	1134	21	10.5	Well water
5	33°33’1.05” N	53°52’23.30” E	955	22	8.7	Ground water
6	33°33’44.01” N	53°50’23.63” E	1021	21	8.5	Ground water
**Site no.3 (Calkafi mine)**						
7	33°23’58.17” N	54°14’18.91” E	1253	24	8.9	Well water
8	33°26’51.76” N	54° 5’34.70” E	923	24	11	Well water
**Site no.4 (Mesr village)**						
9	34° 2’34.59” N	54°47’45.25” E	850	24	7.28	Well water
10	33°56’41.90” N	55° 1’27.22” E	948	15	7.92	Reservoir
**Site no.5 (Ordib zone)**						
11	33°29’33.80” N	54°55’18.45” E	1081	27	8	Ground water
12	33°32’59.04” N	54°55’58.88” E	1061	43	7.4	Ground water
**Site no.6 (Irakan zone)**						
13	33°58’24.08” N	55° 7’15.04” E	851	15	8.13	Reservoir
14	34° 1’44.81” N	55° 6’13.56” E	984	15	8.04	Reservoir
15	34° 7’51.60” N	55° 6’35.85” E	1016	22	7.77	Well water
16	34°12’25.12” N	55°18’55.40” E	721	30	6.54	Well water
17	34°12’18.67” N	55°19’1.61” E	727	33	6.39	Well water

#### ***Gamma-ray detection system***

The activity of ^226^Ra, ^232^Th, ^40^K and ^137^Cs in the samples were measured using a P-type coaxial HPGe detector. The detector has a resolution of 1.89keV at 1.33MeV of ^60^Co. The detector was maintained in a vertical position and shielded by 10 cm thick lead wall, 2 mm cadmium and 3 mm copper to reduce background radiation [10]. Spectrum acquisition was done using the computer software MAESTRO with a multi-channel analyzer (4096-channel) and spectrum analysis was done by using the OMNIGAM software. The reference material IAP-mixed gamma water standard sources (Institute of Atomic Energy POLATOM, Radioisotope Center) containing ^241^Am, ^137^Cs and ^152^Eu were used to obtain efficiency curve. The absolute photopeak efficiencies were determined using following polynomial fit:

(1) $$\begin{array}{ll} \epsilon = & a+\text{blnE}+c{\left(\text{lnE}\right)}^{2}+d{\left(\text{lnE}\right)}^{3}+e{\left(\text{lnE}\right)}^{4}\\ & \;+f{\left(\text{lnE}\right)}^{5}+g{\left(\text{lnE}\right)}^{6} \end{array}$$

Where the constant values a, b, c, d, e, f and g are -0.0004 ± 0.0001, -0.02 ± 0.004, -0.01 ± 0.0006, 0.0001 ± 0.00004, 0.0003 ± 0.00005, 0.000000008 ± 0.000000001 and -0.00000006 ± 0.000000001, respectively.

A wide range of different gamma-ray energy transition lines ranging from about 100 keV up to 1765 keV, associated with the decay products of the ^226^Ra, ^232^Th and ^40^K. The known photopeak lines with background subtraction were used to determine ^226^Ra, ^232^Th and ^40^K. The counting time of sample spectra was also 24 hours.

The activities were measured using following relationship.

(2) $$A=\frac{N}{t\times m\times p\times \epsilon \left(E\right)}$$

where N, t, m, p and ϵ (E) are net area counts, time, intensity, weight of sample and absolute photopeak efficiency at specific energy, respectively [11]. The specific activity of ^226^Ra was evaluated from gamma-ray lines of ^214^Bi at 609.3, 1120.3 and 1764.5keV and ^214^Pb at 295 and 351keV, while the specific activity of ^232^Th was evaluated from gamma-ray lines of ^228^Ac at 338.4, 911.1 and 968.9keV. The specific activity of ^40^K and ^137^Cs was determined from its 1460.8 and 661.6keV gamma-ray lines [12]. The minimum detectable activity for each radionuclide was 0.54 mBq/L for ^226^Ra, 0.21 mBq/L for ^232^Th and 0.01 mBq/L for ^40^K.

### Results and discussion

**Figure 3:**
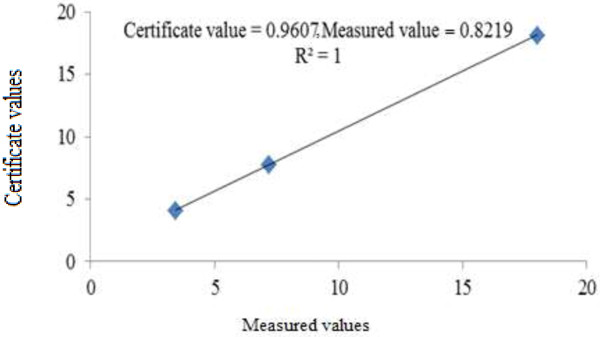
Correlation coefficient for the liner regression between the measured and reference concentrations.

The concentrations of radionuclides of ^241^Am, ^137^Cs and ^152^Eu measured in standard reference material (POLATOM) and results are presented in Table 2. The measured concentrations of ^241^Am, ^137^Cs and ^152^Eu had high consistent with the certificate ones, the correlation coefficient for the liner regression between the measured and reference concentrations was 1 as shown in Figure 3.

**Table 2:** **Comparison of radionuclide concentrations of **^**241**^**Am**, ^**137**^**Cs and **^**152**^**Eu in POLATOM standard reference material**

**Radionuclide**	**Reference activity (kBq)**	**Determined activity (kBq)**	**Determined activity (Bq/L)**
**Am-241**	18.13 ± 0.004	18.03 ± 0.004	0.49±0.004
**Cs-137**	7.77 ± 0.002	7.18 ± 0.002	0.21±0.002
**Eu-152**	4.07 ± 0.001	3.42 ± 0.001	0.11±0.001

#### ***Radioactivity characterization of the subground waters***

**Figure 4:**
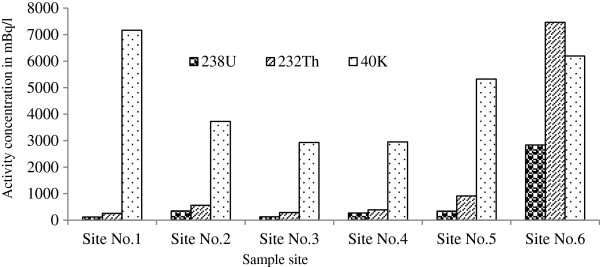
**Distribution of activity concentrations of **^**226**^**Ra, **^**232**^**Th and **^**40**^ **K in water samples.**

Radioactivity levels of ^226^Ra, ^232^Th and ^40^K in the water samples collected from different parts of the studied area are presented in Table 3. As shown in Table 3, activity concentrations in the water samples are in the range of 120 ± 30 - 2836 ± 274 mBq/L for ^226^Ra; 257 ± 39 - 7465 ± 607 mBq/L for ^232^Th and 2930 ± 490 - 7168 ± 1067 mBq/L for ^40^K. The maximum activity concentration of ^226^Ra and ^232^Th is found on site No.6 (Irakan Zone) which may be related to the geological structure of the region. The maximum activity concentration of ^40^K is found in site No. 1 (Talmessi mine). Potassium activity varied widely with CV (Coefficient Variation) =38% due to heterogeneous soil characteristics of this area [13]. The activity concentrations of ^226^Ra and ^232^Th in our samples are compared with the UNSCEAR reference mean values which are 1 mBq/L and 0.05 mBq/L in United States, respectively [14]. The activity concentrations of ^226^Ra and ^232^Th in the studied area are much more than the UNSCEAR reference values in the United States. Comparison of radioactivity of waters with other desertic areas has been done. The concentration of radioactivity of ^226^Ra in Arabia, Nigeria and Pakistan was reported between < MDA – 2500 mBq/L (with an average 1810 mBq/L), <MDA – 5400 mBq/L (with an average 1200 mBq/L) and 11300 mBq/L, respectively [1,5,6]. The mean concentration of ^226^Ra in Anarak-Khour is lower than the average concentration of it in Arabia, Nigeria and Pakistan and also is lower than its minimum concentration in Egypt. The concentration of radioactivity of ^232^Th was reported between < MDA and 3300 mBq/L (with an average 1470 mBq/L) in Arbia, < MDA and 6200 mBq/L (with an average 1600 mBq/L) in Nigeria and 5200 mBq/L in Pakistan. The mean concentration of radioactivity of ^232^Th in Arabia, Nigeria and our studied area is almost identical. The range of concentration of radioactivity of ^40^K was reported between < MDA and 339200 mBq/L (with an average 17596 mBq/L) in Arabia, < MDA and 50900 mBq/L (with an average 25100 mBq/L) in Nigeria. In Pakistan, the mean concentration of ^40^K about 140900 mBq/L was reported. Moreover, the results are compared to the World Health Organization proposed the following guidance levels for the activity concentration in drinking water (10000 mBq/L for ^226^Ra and 1000 mBq/L ^232^Th) [15]. The measured activities of ^226^Ra in the samples did not exceed the guidance level recommended by WHO (World Health Organization). For ^232^Th, Site No.6 had higher activity concentrations (7465 mBq/L >1000 mBq/L) compared to guidance levels recommended by WHO for drinking water. Figure 4 shows the distribution of ^226^Ra, ^232^Th and ^40^K activity concentrations of water samples. As shown in Figure 4, activity concentrations of ^232^Th were higher in water samples than ^226^Ra. In the waters in general, the concentration of ^232^Th was found to be higher than that of the ^226^Ra because ^226^Ra may be created on the surface of the host rock or ejected into the aqueous phase via alpha recoil. Both processes would affect in a depletion of ^226^Ra relative to ^232^Th. Additionally, ^226^Ra is more soluble and mobile in ground water than ^232^Th and may leach from the host rock, independent of the place of creation. There is a relationship between the activity concentration of ^226^Ra and ^232^Th with R square value 0.99 from our sampling site. This shows in all sampling sites the ratio of ^232^Th to ^226^Ra is more than one.

**Table 3:** **Average activity concentration of **^**226**^**Ra, **^**232**^**Th and **^**40**^ **K in water sampling sites**

	**Concentration (mBq/L)**		
	^**226**^**Ra**	^**232**^**Th**	^**40**^**K**
**Site no.1**	120 ± 30	257 ± 39	7168 ± 1067
**Site no.2**	350 ± 54	562 ± 61	3727 ± 577
**Site no.3**	128 ± 30	287 ± 44	2930 ± 490
**Site no.4**	270 ± 49	390 ± 80	2951 ± 496
**Site no.5**	341 ± 63	914 ± 138	5325 ± 837
**Site no.6**	2836 ± 274	7465 ± 607	6196 ± 670
**Average**	674	1646	4716
**Stdev**	1064	2861	1781
**CV (%)**	158	174	38

#### ***Radiological risk assessment***

The absorbed dose rates (D) due to gamma radiation in air at 1m above the ground level, assuming uniform distribution of the naturally occurring radionuclides (^226^Ra, ^232^Th and ^40^K) was calculated based on guidelines is given by UNSCEAR (2000) and IRSN reports (2011). The contributions from other naturally occurring radionuclides have been assumed insignificant. Therefore, D in units of nGy.h^-1^ was calculated according to UNSCEAR (2000) as:

(3) $$D\left(\text{nGy}/h\right)=0.462A_{\mathrm{Ra}}+0.621A_{\mathrm{Th}}+0.0417A_{K}$$

The annual effective outdoor dose rate in units of mSv.year^-1^ was calculated using the following formula [16]:

(4) $$\text{Effectivedoserate}\left(\text{mSv}/\text{year}\right)=1.23\times 10^{‒3}\times \text{Doserate}$$

Radium equivalent activity is an index that has been used to represent the specific activities of ^226^Ra, ^232^Th and ^40^K by a one quantity, which takes into account the radiation hazards associated with them. The radium equivalent activity is a weighted sum of activities of the studied natural radionuclides and is based on the assumption that 370Bq/L of ^226^Ra, 259Bq/L of ^232^Th, and 4810Bq/L of ^40^K produce the same gamma radiation dose rate [16]. The maximum value of Ra_eq_ must be less than 370Bq/L for safe use [17]. It is defined as follows:

(5) $${\mathrm{Ra}}_{\mathrm{eq}}=A_{\mathrm{Ra}}+1.43\times A_{\mathrm{Th}}+0.077A_{K}$$

where A_Ra_, A_Th_ and A_K_ are the activity concentrations of ^226^Ra, ^232^Th and ^40^K, respectively.

A widely used hazard index called the external hazard index H_ex_ is defined as follows [13]:

(6) $$H_{\mathrm{ex}}=A_{\mathrm{Ra}}/370+A_{\mathrm{Th}}/259+A_{K}/4810$$

In addition to the external hazard index, radon and its short-lived progeny are also hazardous to the respiratory organs. The internal exposure to radon and its daughter progenies is quantified by the internal hazard index H_in_, which is given by the equation [13]:

(7) $$H_{\mathrm{in}}=A_{\mathrm{Ra}}/185+A_{\mathrm{Th}}/259+A_{K}/4810$$

The calculated total gamma dose rate due to primordial radionuclides varied from 0.29 to 6.102 nGy/h. The average total gamma dose rate was less than the worldwide average of 55 nGy/h [12]. The annual effective dose obtained in the investigated areas ranged from 0.36 to 7.502μSv for the background area. The reported gamma dose rate and annual effective dose in other places, such as Lake Bosumtwi Basin in Ghana [18] is higher than these areas. But, Abo Zaabal in Egypt [19], Niger Delta (Biseni) flood plain lakes [20], Outer Carpathians in Poland [21] is lower or at least equal to the values of this research. If the value of internal or external radiation hazard index is found to be less than unity, then there is no potential internal or external radiation hazard. The external radiation hazard index (H_ex_) in water varied from 0.002 to 0.0376 whereas the internal radiation hazard index (H_in_) in water varied from 0.002 to 0.0448. This indicated that the water of Anarak-khour area were free from the radiation hazards. The absorbed dose rates (D), annual effective outdoor dose rate, radium equivalent activity (Ra_eq_) H_ex_ and H_in_ of our samples are calculated and their results are shown in the Table 4. As seen in this table, H_ex_ and H_in_ of our results are less than unity which shows there is no radiation hazard for the body.

**Table 4:** Calculated average values of absorbed dose rate and annual effective dose, radium equivalent activity, external and internal radiation hazard

	**Absorbed dose rate (nGy/h)**	**Radium equivalent activity (Bq/L)**	**External radiation hazard index (H**_**ex**_**)**	**Internal radiation hazard index (H**_**in**_**)**
**Site no.1**	0.525	1.015	0.003	0.003
**Site no.2**	0.665	1.415	0.004	0.004
**Site no.3**	0.290	0.625	0.002	0.002
**Site no.4**	0.490	1.035	0.003	0.003
**Site no.5**	0.895	1.930	0.005	0.006
**Site no.6**	6.102	13.770	0.038	0.045

## Conclusion

Our estimate of water radioactivity concentration on Anarak-khour area in central of Iran is done using gamma-ray spectrometry. The maximum activity concentration of ^226^Ra and ^232^Th is found in Irakan Zone. The maximum activity concentration of ^40^K is found in Talmessi mine. The measured activities of ^226^Ra in the samples did not exceed the guidance level recommended by WHO but the measured activities of ^232^Th in Irakan Zone exceeded. The calculated total gamma dose rate varied from 0.29 to 6.102 nGy/h. The annual effective dose obtained from 0.36 to 7.502μSv for the background area. The internal radiation hazard index (H_in_) in water varied from 0.002 to 0.0448. The parameters of absorbed dose rate, annual effective dose, radium equivalent activity, external radiation hazard index and internal radiation hazard index is calculated and their results showed there is no potential internal radiation hazard. This study can be followed by analyzing the deep soil and plants of the studied area. Moreover, because there are a lot of people who physically are impaired, the birth rate of children with defects should be compared with the radiounuclide concentrations in soils, waters and plants in every few years. Our results will contribute to the data base of this area in future. Then it is necessary that after operating the disposal site of nuclear waste all environment samples of the studied area should be performed every year and compared with our results.

## Competing interests

The authors declare that they have no competing interests.

## Authors’ contributions

EE carried out the sampling, participated in the experimental analysis in laboratory. MRA participated in the analysis of the data and drafted the manuscript. MM conceived of the study, and participated in sampling. HB participated in geology part. All authors read and approved the final manuscript.

## References

[B1] Fakeha A Hamidalddin S Alamoudy Z Al-Amri MA Concentrations of natural radioactivity and their contribution to the absorbed dose from water samples from the Western Province, Saudi Arabia JKAU: Sci 2011 23 2 17 30

[B2] Selçuk Zorer Ö Ceylan H Doğru M Gross alpha and beta radioactivity concentration in water, soil and sediment of the Bendimahi River and Van Environ Monit Assess 2009 148 39 46 10.1007/s10661-007-0137-x 18193334

[B3] Abdi MR Kamali M Vaezifar S Distribution of radioactive pollution of ^226^Ra, ^232^Th, ^40^ K and ^137^Cs in northwestern coasts of Persian Gulf, Iran Mar Pollut Bull 2008 56 751 757 10.1016/j.marpolbul.2007.12.010 18241891

[B4] Sherwood Lollar B Environmental Geochemistry, Volume 9: Treatise on Geochemistry 2005 Amsterdam: Elsevier

[B5] Fasunwon OO Alausa SK Odunaike RK Alausa IM Sosanya FM Ajala BA Activity concentrations of natural radionuclide levels in well waters of Ago Iwoye Nigeria Iran J Radiat Res 2010 7 4 207 210

[B6] Fatima I Zaidi JH Arif M Tahir SNA Measurement of natural radioactivity in bottled drinking water in Pakistan and consequent dose estimates Radiat Prot Dosim 2007 123 2 234 240 10.1093/rpd/ncl093 16877468

[B7] Ramezani J Tucker R The Saghand region, Central Iran: U-Pb geochronology, petrogenesis and implications for Gondwana tectonics Am J Sci 2003 303 622 665 10.2475/ajs.303.7.622

[B8] Bagheri H Moore F Alderton DHM Cu-Ni-Co-As (U) mineralization in the Anarak area of Central Iran J Southeast Asian Earth Sci 2006 29 651 665

[B9] Abbas MI HPGe detector photopeak efficiency calculation including self absorption and coincidence corrections for Marinelli beaker sources using compact analytical expressions Appl Radiat Isot 2001 54 761 768 10.1016/S0969-8043(00)00308-0 11258525

[B10] Debertin K Helmer RG Gamma and X-ray spectrometry with semiconductor detectors 1988 Amsterdam: Elsevier

[B11] Faghihian H Rahi D Mostajaboddavati M Study of natural radionuclides in Karun river region J Radioanal Nucl Chem 2012 292 711 717 10.1007/s10967-011-1496-x

[B12] Abdi MR Hassanzadeh S Kamali M Raji HR ^226^Ra, ^232^Th, ^40^K and ^137^Cs activity concentrations along the southern coast of the Caspian Sea, Iran Mar Pollut Bull 2009 58 658 662 10.1016/j.marpolbul.2009.01.009 19261302

[B13] Kinyua R Atambo VO Ongeri RM Activity concentrations of ^40^K, ^232^Th, ^226^Ra and radiation exposure levels in the Tabaka soapstone quarries of the Kisii Region, Kenya African J Environ Sci Tech 2011 5 682 688

[B14] UNSCEAR Sources and Effects of Ionizing Radiation (Report to the General Assembly) 2000 New York: United Nations Publication

[B15] WHO Guidelines for drinking water quality 2004 Geneva: IWA Publishing

[B16] Farai IP Ademola JA Radium equivalent activity concentrations in concrete building blocks in eight cities in Southwestern Nigeria J Environ Radioact 2005 79 119 125 10.1016/j.jenvrad.2004.05.016 15603902

[B17] Abbady AGE Estimation of radiation hazard indices from sedimentary rocks in Upper Egypt Appl Radiat Isot 2004 60 111 114 10.1016/j.apradiso.2003.09.012 14687644

[B18] Adu S Darko EO Awudu AR Adukpo OK Emi-Reynolds G Obeng M Otoo F Faanu A Agyeman LA Mensah CK Hasford F Ali ID Agyeman BK Kpordzro R Preliminary Study of Natural Radioactivity in the Lake Bosumtwi Basin Res J Environ Earth Sci 2011 3 463 468

[B19] Morsy Z El-Wahab MA El-Faramawy N Determination of natural radioactive elements in Abo Zaabal, Egypt by means of gamma spectroscopy Ann Nucl Energy 2012 44 8 11

[B20] Agbalagba EO Onoja RA Evaluation of natural radioactivity in soil, sediment and water samples of Niger Delta (Biseni) flood plain lakes, Nigeria J Environ Radioact 2011 102 667 671 10.1016/j.jenvrad.2011.03.002 21514983

[B21] Kozłowska B Walencika A Dordaa J Przylibskib TA Uranium, radium and 40Kisotopes in bottled mineral waters from Outer Carpathians, Poland Radiat Meas 2007 42 1380 1386 10.1016/j.radmeas.2007.03.004

